# Valentino’s syndrome: a bizarre clinical presentation

**DOI:** 10.1093/jscr/rjad035

**Published:** 2023-02-06

**Authors:** Dennis Machaku, Mujaheed Suleman, Elias Mduma, Mugisha Nkoronko

**Affiliations:** General Surgery, Kilimanjaro Christian Medical Center, Kilimanjaro, Tanzania; Kilimanjaro Christian Medical University College, Kilimanjaro, Tanzania; General Surgery, Kilimanjaro Christian Medical Center, Kilimanjaro, Tanzania; Kilimanjaro Christian Medical University College, Kilimanjaro, Tanzania; General Surgery, Kilimanjaro Christian Medical Center, Kilimanjaro, Tanzania; Kilimanjaro Christian Medical University College, Kilimanjaro, Tanzania; General Surgery, Kilimanjaro Christian Medical Center, Kilimanjaro, Tanzania

## Abstract

A perforated peptic or duodenal ulcer may cause an unusual expression of right lower quadrant pain. In Valentino’s syndrome, the chemical fluid from the ulcer flows via the right paracolic gutter to the right iliac fossa, causing peritoneal irritation and chemical appendicitis which will mimic pain in the right lower quadrant. We report a case of a 23-year-old male patient who presented with cramping lower abdominal pain with fevers and vomiting. His pain was mostly in the right lower quadrant and radiated to his back. A perforation-related pneumoperitoneum was found on a computed tomography scan, along with an accumulation of fluid in the abdomen and thickening of the pyloric antrum. Valentino’s syndrome’s aberrant clinical picture mimicking acute appendicitis is a pathognomonic presentation of the disease. Right lower abdominal pain should also prompt the scrutiny of atypical differentials, such as perforated ulcers. Physicians need to manage these patients with a high index of suspicion.

## INTRODUCTION

It has long been stipulated that acute appendicitis is one of the most prevalent clinical manifestations of right lower quadrant pain. It is the most frequent complaint for patients with acute abdomen at the emergency unit [1]. In addition, physicians keep an eye out for various differential diagnoses for patients who present with right lower quadrant pain and elicit multiple etiologies that mimic acute appendicitis, making it onerous to make a diagnosis. There are a few uncommon disorders, such as ruptured ectopic pregnancy, perforated peptic ulcer, ovarian torsion and others, that might coincide with the clinical presentation of acute appendicitis [2]. Valentino’s syndrome involves the presentation of a perforated peptic ulcer. The chemical fluid from the ulcer streams along the right paracolic gutter to the right iliac fossa, causing peritoneal irritation and consequent chemical appendicitis [3]. In patients presenting with right lower quadrant pain, including Valentino’s syndrome as an uncommon differential is critical [4].

This case report describes a patient who reported to our facility with acute lower abdominal discomfort that was accompanied by low-grade fevers and vomiting. The study will also delineate the management course and related literature.

## CASE PRESENTATION

**Figure 1:**
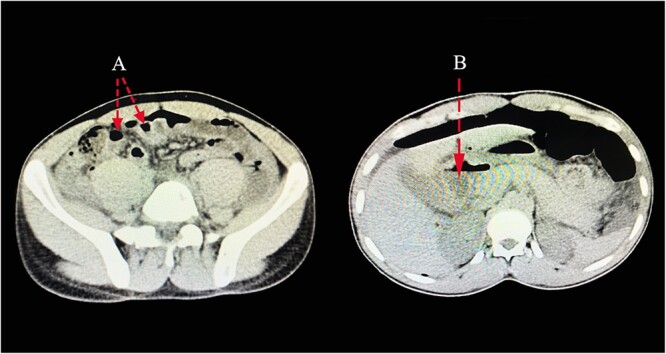
CT scan images pointing out presence of pneumoperitoneum related to a perforation (**A**), with free fluid collection in the abdomen (**B**).

**Figure 2:**
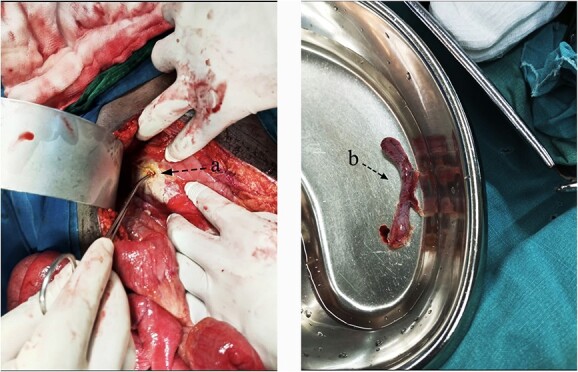
Intraoperative findings of a pinpoint perforation at the anterior aspect of the pyloric antrum (**A**); a consequent appendectomy was done, with the imaging (**B**) showing the removed appendix.

A 23-year-old male patient was sent to our emergency department from a nearby hospital. He arrived with a week’s history of cramping lower abdomen discomfort, mostly in his right lower quadrant and radiating to his back, that began gradually and worsened with time. During the course, the illness was associated with recurrent low-grade fevers and a single episode of vomiting that contained previously consumed meals. His bowel habits, however, remained unchanged. He was clinically stable when he arrived, with blood pressures of 124/73 mmHg, pulse rates of 87 bpm and saturating well on room air. He had a regular abdominal contour that moved in sync with his breathing. On palpation, there was mild discomfort with guarding along his right and left iliac as well as umbilical aspect. On percussion across the epigastric and umbilical region, there was a normal tympanic tone, and typical bowel sounds were appreciated. His full blood count indicated that he had increased neutrophils, but his electrolytes were normal. An NGT placement was commenced. A provisional of acute appendicitis was obtained along with differential diagnoses of a ruptured appendix with peritonitis and perforated peptic ulcer. An abdominal computed tomography (CT) scan with contrast revealed pneumoperitoneum related to a perforation, with free fluid collection in the abdomen and a thickening of the pyloric antrum measuring ~1.3 cm (Fig. 1). Abdominal USS identified symptoms of an appendix rupture. An explorative laparotomy was imminent. Following a midline abdominal incision, the abdomen was incised in layers. There was a liter of turbid peritoneal fluid and several immature adhesions, an inflamed retrocecal appendix and multiple fibrin exudates, which were observed in the cecum and right paracolic gutter, with stomach contents reaching the appendiceal aspect. A 0.5 × 0.2-cm perforation was discovered on the pyloric antrum at the anterior region of the lesser curvature. An appendectomy was done after adhesion lysis and a tissue sample was taken for histology. Modified Graham’s technique was used to repair the perforation. Abdominal lavage with copious amounts of fluids was done, drains were placed and the fascia and skin were sealed with Nylon sutures (Fig. 2). Post-operatively, the patient had a quick recovery and was discharged after 5 days. Subsequently, the patient’s follow-up assessments were unremarkable.

## DISCUSSION

The expansive clinical presentations of the acute abdomen often require careful observations. Patients presenting with typical signs of right lower quadrant pain may occasionally pose a challenge in eliciting definite diagnoses due to the majority of differentials accompanying the presentation. The atypical manifestation of Valentino’s syndrome mimicking acute appendicitis is often missed during initial observations by physicians because of the peculiar pathognomonic presentation of the disease. This syndrome was named after a film actor named Rudolph Valentino and was described after he experienced the signs and symptoms of appendicitis. He underwent an appendectomy that did not alleviate his symptoms. He then developed overt peritonitis and multi-organ failure, which led to his death. A perforated peptic ulcer was confirmed during his autopsy as the reason for his death [5]. Fluid leaking from a perforated gastric or duodenal ulcer may induce peritonitis if it drains down the right paracolic gutter. It may spread to the appendix, causing chemical irritation that mimics acute appendicitis and culminating in Valentino’s syndrome [6]. The presence of pneumoperitoneum is often formed by the intraperitoneal perforation of the stomach or first part of the duodenum, whereas pneumo-retroperitoneum (typical in the right kidney, ‘veiled right kidney sign’) is caused by a retroperitoneal perforation [7]. The most reliable sign of appendicitis frequently manifests as epigastric or periumbilical pain that progressed to the RLQ [8].

According to the initial presentations of our patient, we judicially went on with the initial delineation of acute appendicitis. A typical picture of lower abdominal tenderness with guarding along his right and left iliac as well as the umbilical aspect showed a pathognomonic presentation of consequent chemical peritonitis following an appendiceal rupture. Concurrent imaging modalities were crucial in evaluating a full-scale pathological presentation of the viscera. The confirmation of perforation on the stomach or duodenum is mostly made intraoperatively on surgical exploration and without adequate imaging analysis. On an erect chest X-ray, free peritoneal air may be identified as the air under the diaphragm. In ultrasonography or intraoperatively, a fluid collection is encircling the appendix as the gastric fluid courses through the right paracolic gutter into the right iliac fossa. In the context of acute appendicitis with epigastric pain, a CT scan is the suggested imaging investigation for the diagnosis of Valentino’s syndrome. [9]. A proposed CT scan may reveal the presence of free fluid with exudates, with features suggestive of a perforation [10, 11]. The role of laparoscopic surgery should be enunciated in cases of diagnostic dilemma. Delayed diagnosis and management can have calamitous complications, including morbidity and mortality in these patients [12]. These perforations are managed with a simple closure. Simple closures, with or without Graham’s technique, are surgical procedures that can be utilized. In exploratory laparoscopic surgery, simple closures are also employed. The *H. Pylori* eradication is routinely the next step after the operation [3, 13].

It is evident from our patient that a perforated ulcer might uncommonly mimic acute appendicitis. When patients present with right lower quadrant pain, the majority of physicians stress the prospect of acute appendicitis. In the majority of cases, conditions may be delineated by completing a detailed clinical history, physical examination and imaging studies for the patient. But the prevalence of right lower abdominal pain should prompt the scrutiny of atypical differential diagnoses, such as perforated ulcers. It is essential to assess the possibility of a perforated ulcer in the context of bile-stained or turbid peritoneal fluid in a setting of an appendectomy.
